# Drug Selection and Posology, Optimal Therapies and Risk/Benefit Assessment in Medicine: The Paradigm of Iron-Chelating Drugs

**DOI:** 10.3390/ijms242316749

**Published:** 2023-11-25

**Authors:** George J. Kontoghiorghes

**Affiliations:** Postgraduate Research Institute of Science, Technology, Environment and Medicine, Limassol 3021, Cyprus; kontoghiorghes.g.j@pri.ac.cy; Tel.: +357-26272076

**Keywords:** iron overload, iron chelation therapy, thalassemia, neurodegenerative diseases, optimal dose protocols, aspirin, drug efficacy, drug toxicity, pharmacology

## Abstract

The design of clinical protocols and the selection of drugs with appropriate posology are critical parameters for therapeutic outcomes. Optimal therapeutic protocols could ideally be designed in all diseases including for millions of patients affected by excess iron deposition (EID) toxicity based on personalised medicine parameters, as well as many variations and limitations. EID is an adverse prognostic factor for all diseases and especially for millions of chronically red-blood-cell-transfused patients. Differences in iron chelation therapy posology cause disappointing results in neurodegenerative diseases at low doses, but lifesaving outcomes in thalassemia major (TM) when using higher doses. In particular, the transformation of TM from a fatal to a chronic disease has been achieved using effective doses of oral deferiprone (L1), which improved compliance and cleared excess toxic iron from the heart associated with increased mortality in TM. Furthermore, effective L1 and L1/deferoxamine combination posology resulted in the complete elimination of EID and the maintenance of normal iron store levels in TM. The selection of effective chelation protocols has been monitored by MRI T2* diagnosis for EID levels in different organs. Millions of other iron-loaded patients with sickle cell anemia, myelodysplasia and haemopoietic stem cell transplantation, or non-iron-loaded categories with EID in different organs could also benefit from such chelation therapy advances. Drawbacks of chelation therapy include drug toxicity in some patients and also the wide use of suboptimal chelation protocols, resulting in ineffective therapies. Drug metabolic effects, and interactions with other metals, drugs and dietary molecules also affected iron chelation therapy. Drug selection and the identification of effective or optimal dose protocols are essential for positive therapeutic outcomes in the use of chelating drugs in TM and other iron-loaded and non-iron-loaded conditions, as well as general iron toxicity.

## 1. Introduction

The pharmacological aspects of medicinal drugs including the selection and application of effective dose protocols are critical for the treatment of patients in all diseases. For example, individual variations in drug responses [1], differences among populations [2], different drug formulations [3], single or multiple drug administration [4] and different routes of drug administration [5] all have different effects on treatments. Similarly, the need for optimal, target-specific therapies including drug combinations as well as other parameters will also effect overall treatments [6,7,8,9,10]. The risk/benefit assessment of drug posology selection and effective use is a continuous developmental process even after drug registration and during post-marketing surveillance [11,12,13,14,15]. Following drug discovery, the identification of appropriate dose protocols, diagnostic methods and toxicity effects including evaluation of changes in target organ(s) affected by drug toxicity is an essential parameter for the long-term assessment and development of drugs.

Despite evaluation of drug posology selection, there is variation among individuals in drug response as a result of many different parameters associated with different factors including genomic [16,17,18,19,20], immunological [20,21], pharmacological [22,23] and toxicological variations [24,25], as well as interactions with drugs, dietary substances, etc. Similarly, there are also differences among individuals in drug response as a result of many other limitations such as underlying disease pathological complications, variations in organ functioning, idiosyncratic reactions, accessibility to effective drug posology due to high cost and other variations [22,23,24,25,26].

In this context and considering all the different variables, every effort should be made to design optimal therapeutic protocols for all diseases, which could be individualised for each patient within the context of personalised medicine. In particular, this approach should be considered where variations in drug response are measurable, such as, for example, drug absorption, distribution, metabolism and elimination (ADME) parameters [26,27,28,29] and also for toxicity such as drug allergy or other drug reactions or interactions [29,30,31,32,33,34]. Furthermore, the availability of alternative drugs should also be an option for patients with low response and/or toxicity to a particular leading drug for a specific disease [35,36,37,38,39,40]. 

A major advancement in optimizing drug protocols with increasing therapeutic successes in many diseases has been the introduction of drug combination therapies [13,22,41,42,43,44,45]. This approach is in most cases almost exclusively based on academic initiatives and is not generally regulated by the drug regulatory authorities, nor does it usually involve the manufacturers of each of the drugs in the combination. Similarly, a major drawback in drug development and therapeutic outcomes is also that investigations on drug toxicity and introduction of possible antidotes mostly rely exclusively on academic initiatives and not on the drug manufacturers [46,47,48,49,50]. 

The challenges in drug design, development and use are continuously expanding. In particular, there are many and different regulatory and other approaches for the introduction of drug treatments in millions of patients with chronic and untreatable diseases [51,52,53,54,55,56], in emergency medicine and also in orphan diseases [57,58,59,60,61,62], where drug cost is an important parameter [59,60,61,62]. Many examples of such approaches include the call for emergency medicines in the recent COVID-19 pandemic [63] and the expanding area of drug repurposing [64] in many other untreatable diseases [59,60,61,62,63,64,65,66].

Similarly, new advances in drug development include the introduction of improved drug posology [67,68,69], different routes of administration [70,71], new improved formulations, nanomedicines and other drug carriers, and drug combinations [71,72,73,74]. In the meantime, it should be noted that millions of patients choose to be treated in alternative medicine clinics using non-conventional treatments including a wide range of folk medicines, nutraceuticals and alternative medicinal drugs and combinations [75,76,77,78,79]. 

One major area of drug investigations involves essential, xenobiotic, diagnostic and theranostic metals. The use of different metals in medicine is accomplished by metal carriers, which are mainly chelating agents [80]. Metal-chelating drugs are widely used in medicine mostly for drug detoxification but also as metal complexes for increasing essential metal ion absorption and also for therapeutic, diagnostic and theranostic applications. Similarly, many drugs and dietary components have metal-binding potential and their interactions with metals and other metal-binding drugs affect their therapeutic and biological activities [80].

Iron-chelating drugs are widely used for the elimination of excess iron in the treatment of many categories of chronically red-blood-cell (RBC)-transfused patients and especially thalassemia major (TM) patients. Thalassemia major is classified as an orphan disease and iron-chelating drugs belong to the orphan drug category [62]. Many other categories of patients in addition to TM are affected by excess iron deposition (EID) and other forms of iron toxicity. The mechanistic insights in diseases related to EID and toxicity from the molecular, cellular and tissue level to clinical implications have been recently reviewed [81]. 

The aim of this review is to identify the major factors and parameters associated with the design, development and use of iron-chelating drugs in medicine including drug selection, posology and metabolic aspects for maximum efficacy and low toxicity. In particular, the risk/benefit assessment of the application of iron-chelating drugs in different clinical conditions and for new clinical targets of iron toxicity will be discussed including examples such as the complete elimination of EID in the treatment of iron overload in TM, as well as some of the drawbacks in the use of iron-chelating drugs, including toxicity and suboptimal posology effects in neurodegenerative diseases. 

## 2. Design and Developmental Aspects of Chelating Drugs for the Elimination of Excess Iron

**Figure 1:**
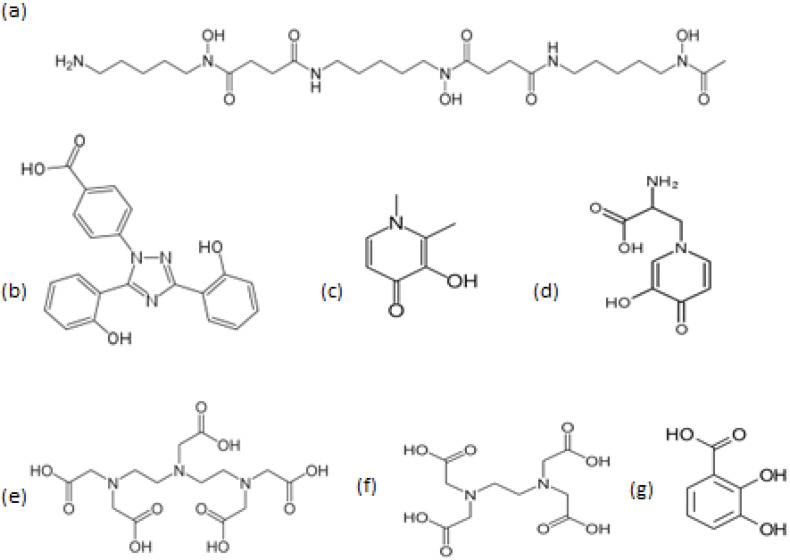
The structure of the iron-chelating drugs and other chelators with clinical effects. The iron-chelating drugs deferoxamine (**a**), deferasirox (**b**) and deferiprone (**c**) are widely used for the treatment of transfusional iron overload. Mimosine (**d**) and 2,3-dihydroxybenzoic acid (**g**) are iron-binding phytochelators with clinical potential. Diethylenetriaminepentacetic acid (DTPA) (**e**) and ethylenediaminetetracetic acid (EDTA) (**f**) are synthetic iron-binding drugs used for general metal detoxification.

There are three iron-chelating drugs, namely, deferoxamine (DF), deferiprone (L1) and deferasirox (DFRA), which are widely used in thousands of patients worldwide mainly for the treatment of transfusional iron overload in TM and other diseases [82,83,84,85,86]. The general aim of these three iron-chelating drugs is the removal of excess iron, which is accumulated in the body as a result of chronic RBC transfusions; it is toxic and can be fatal unless removed. Both L1 and DFRA are orally active, whereas DF is orally inactive and has to be administered subcutaneously and sometimes intravenously to be effective (Figure 1). The iron-binding properties and effects of the iron-chelating drugs and a number of naturally occurring phytochelators such as mimosine and 2,3-dihydroxybenzoic acid or synthetic chelating drugs such as EDTA and DTPA have also been previously introduced and compared to the chelating drugs [62,66,80,81] (Figure 1).

More specifically, the physicochemical, pharmacological, toxicological and other properties of DF, L1 and DFRA have been previously reviewed [87]. A summary of some of the chemical, physicochemical, biological, pharmacological, toxicological and clinical properties of the iron-chelating drugs DF, L1 and DFRA is provided in Table 1.

There are many stages historically in relation to the development of iron-chelating drugs for TM. All TM patients worldwide died untreated until the 1940s. The major initiatives for treatment began with the successful introduction of RBC transfusions in some TM patients in the mid-1940s, which increased the hope for longer survival in comparison to the early mortality of within 1–3 years from birth of non-RBC-transfusion-treated patients [88]. However, despite the fact that survival in TM has substantially increased with the introduction of RBC transfusions, it soon became apparent that the build-up of excess iron in many organs and the associated toxicity was also life-threatening and could be fatal unless the excess iron was removed. 

Further breakthrough developments were later reported with the introduction of DF in the early 1960s, where the prospects of the excess toxic iron removal treatment in TM increased once again [89]. The successful first application of the chelating drug DF for the removal of iron and the treatment of iron overload in TM became a reality. However, the success was limited and short-lived because of the copious administration procedure and the low efficacy of the daily intramuscular injections of DF initially used [90,91]. A substantial improvement in iron excretion was observed following long-term administration, e.g., 24 h intravenous infusion of DF in comparison to its bolus administration [92,93]. In particular, the introduction of the daily 8–12 h subcutaneous administration of DF using a mechanical pump was more feasible and effective for increasing iron excretion and also for long-term use, all of which helped to increase further the overall survival of TM patients [94,95,96]. This improvement in iron chelation therapy was originally available only to some patients in developed countries, where the high cost of the mechanical pump and DF was affordable. Similarly, further improvements in the subcutaneous administration of DF was observed in the last 10–15 years using a 6–48 h elastomeric pump administration, which is less copious and more efficient in increasing iron excretion than the 8–12 h mechanical pump administration [97].

In the meantime, the research efforts for designing or identifying an effective and preferably an orally active iron-chelating drug were initiated well before the introduction of DF. These efforts continued following the introduction of DF in order to complement or overcome the low efficacy, toxicity and low compliance of DF in many TM patients. In this context, hundreds of synthetic and naturally occurring chelators were tested in different experimental models for the prospect of replacing DF with a more effective and/or orally active chelating drug. 

Only a few of the experimental chelators tested in vitro and in vivo have reached the stage of clinical testing for use in TM. Among these are several naturally occurring chelators such as the phytochelator 2,3-dihydroxybenzoic acid, which was administered to TM patients at 25 mg/kg four times a day for a year but was not effective [98,99] and rhodotorulic acid, which was administered parenterally but was found to be toxic [100]. Many more synthetic chelating agents were also tested, including the widely available and used ethylenediaminetetraacetic acid (EDTA) and diethylenetriaminepentaacetic acid (DTPA), which however increased the excretion of essential metals and were abandoned [90,101,102,103], as well as their derivatives ethylenediaminehydroxyphenylacetic acid and N, N1-bis (o-hydroxybenzyl) ethylenediamine-N,N1-diacetic acid, which were also abandoned [104,105,106]. The disappointing results continued with newly designed chelators such as pyridoxalisonicotinoylhydrazone, salicylhydroxamic acid and cholylhydroxamic acid [105,107,108]. Furthermore, several experimental drugs including the anticancer experimental drug 5-hydroxy-2-formylpyridinethiosemicarbazone have also been clinically tested; that latter drug was found to cause substantial increase in iron excretion in non-iron-loaded cancer patients [109,110]. Overall, almost all the above experimental chelators were abandoned following clinical testing because of insufficient efficacy in iron excretion or toxicity or for both of these reasons.

The above chelators were clinically tested prior to the development of L1. Similarly, several other chelators were tested clinically following the first clinical trials of L1, but these were also later abandoned because of similar efficacy or toxicity reasons, as well as a lack of interest from the pharmaceutical industry. These include 1,2-diethyl-3-hydroxypyrid-4-one, 1-ethyl-2-methyl-3-hydroxypyrid-4-one and 1-allyl-2-methyl-3-hydroxypyrid-4-one [111,112,113,114,115].

The historical aspects of the design, development and use of L1 and DFRA were also recently reviewed [111,112,116,117]. In brief, the design and most of the developmental work including the initial clinical trials of L1 were based on academic initiative and sponsored by the UK Thalassaemia Society, which is a charity organization [117]. In contrast, the development of the latest oral iron-chelating drug, DFRA, was based on a pharmaceutical company initiative, which was from the same company as the previous proprietor of DF until its patent expiration [111,117]. 

Despite the improved therapeutic outcome with the introduction of the three iron-chelating drugs (DF, L1 and DFRA), the treatment in most cases of TM and other iron-loaded patients is not completely satisfactory and the patients affected by iron toxicity struggle daily to reach and maintain non-toxic levels of excess iron. Similarly, only a few TM patients succeeded in reaching and maintaining normal iron body levels, mainly because of misinformation or lack of experience in physicians regarding the benefits of such treatment to patients. Within this context, the aim of iron chelation therapy needs to be defined and accordingly the design of effective and non-toxic chelation protocols for optimal therapies introduced for TM and other iron-loaded patients [111,117,118]. 

Similar considerations regarding chelation therapy to those of TM apply in the risk/benefit assessment for the use of each of the iron-chelating drugs in non-iron-loaded diseases where EID or other forms of iron toxicity are identified in a particular organ. There are many such cases where iron toxicity is implicated, such as EID in the brain of many neurodegenerative diseases or where other forms of iron toxicity are implicated, for example, in free radical pathology or in the inhibition of a metabolic pathway involving a key iron-containing enzyme. In each of these non-iron-loaded clinical condition cases, different limitations apply in the design and application of drug protocols involving each one of the iron-chelating drugs, which depend on the properties of the drug and the underlying condition of each patient category, as well as other factors affecting individual patients [66]. 

## 3. The Aim of Iron Chelation Therapy in Thalassemia and the Design of Effective Chelation Protocols

Iron chelation therapy can prevent iron overload toxicity and can substantially decrease the associated mortality and morbidity in TM and other categories of regularly RBC-transfused iron-loaded patients [81,82,83,119,120,121]. This goal can be achieved using effective chelating drug protocols, which can cause negative iron balance, where the amount of iron removed from the body is higher than the amount of iron accumulated by the intake of excess iron from RBC transfusions and also from increases in dietary iron absorption [122]. 

The ultimate aim of iron chelation therapy in transfusional iron overload in TM and other refractory anemias is the removal of all excess toxic iron and the maintenance of normal iron store levels [123]. Normal iron levels in TM patients are associated with an overall decrease in morbidity and mortality due to a decrease in cardiac, liver, endocrinologic or other organ damage, which are caused by iron overload toxicity [119,120,121,124,125,126,127,128,129,130,131]. In general, normal iron stores can be characterised by normal physiological range of serum ferritin (350 μg/L>), cardiac T2* (>20 ms) and liver T2* (>6.3. ms) MRI relaxation time levels [81,132].

Iron overload toxicity from chronic RBC transfusions leads to progressive multi-organ damage. In the absence of chelation therapy, TM patients usually die in their teenage years, mainly from congestive cardiac failure caused primarily by cardiac iron overload toxicity [81,119,120,121]. Despite the fact, that mean survival in TM increased following the introduction of subcutaneous DF, the major cause of mortality was again cardiac iron overload in the DF-treated patients [133]. Currently, the use of specific chelation therapy protocols and especially the use of L1 and an L1/DF combination, as well as possible combinations including DFRA, appear to increase further life expectancy and reduce the morbidity of iron-loaded patients to levels approaching those of normal individuals [134,135,136,137,138]. 

All three chelating drugs have exceeded the patent restriction period and are classified as generic pharmaceuticals and belong to the orphan drug category [62]. Deferoxamine has been used parenterally for the treatment of iron-overloading conditions for more than 60 years [89,90,91,92,93,94]. Oral L1 was approved by the regulatory authorities of India in 1995, in Europe and in other countries in 1999 and in the USA in 2011 [112,139]. Oral DFRA has been introduced worldwide since 2005 [140,141]. There are major differences in the efficacy, tolerance, site of action, toxicity profile and cost of the chelating drugs, all of which affect the morbidity and mortality of iron-overloaded patients both in developed and developing countries (Table 1) [62,82,83,84,87]. Despite the wide availability of iron-chelating drugs in developed countries, many iron-loaded TM patients in developing countries are not receiving any treatment because of the high cost of the transfusions and the drugs [62,84]. However, in the last 20–30 years the local manufacturing of cheaper formulations for L1 and DFRA in developing countries such as India, Iran and Thailand has substantially increased the number of TM patients receiving chelation therapy in these and also other neighbouring countries [62,142,143]. In contrast, no substantial change in the price of the three iron-chelating drugs is observed in the developed countries despite the fact that they have all been listed in the generic drug category for many years [62,84]. 

Many factors influence the therapeutic application of iron-chelating drugs in general, with different iron-chelating drugs and doses used among the various countries and clinics worldwide. The recommended and approved range of doses of the iron-chelating drugs in iron-overloaded TM patients and other similar patient categories are 40–60 mg/kg/day for subcutaneous DF, 75–100 mg/kg/day for oral L1 and 20–40 mg/kg/day for oral DFRA (Table 1) [87,122,136]. In most cases, personalised chelation therapies including combination therapies are designed and applied depending on the total body iron load and also the therapeutic targeting of a specific iron-loaded organ. In such cases, therapeutic protocols can be designed ranging from an intensive chelation, such as combinations of DF (40–60 mg/kg/day) and L1 (75–100 mg/kg/day) in heavily transfused iron-loaded patients [144], to much lower doses and frequency of chelating drug administration with intermittent withdrawal of chelation, such as in the cases of TM, thalassemia intermedia (TI) and sickle cell disease (SCD) patients who have achieved normal body iron store levels [87,122,145]. In the latter cases, uncontrolled continuation of chelation therapy may cause iron deficiency and other complications [146]. 

There are many variations in chelation therapy among patients including the rate of RBC transfusions, level of iron overload and organ distribution, as well as pharmacologic and toxicological aspects related to each of the chelating drugs and also in the overall chelation therapy outcome. The therapeutic responses are also related to individual profile differences in the absorption, distribution, metabolism, elimination and toxicity (ADMET) of the drugs [87,147]. The pharmacokinetic and metabolic properties differ among the chelating drugs, with DF rapidly cleared from blood in minutes, L1 in about 6 h and DFRA in more than 19 h. Similarly, L1 causes an increase in iron excretion exclusively in the urine, DFRA exclusively in the feces and DF mostly in the urine and also some in the feces (Table 1) [148,149,150,151].

There are many complexities in the selection of the chelation protocol in each iron-loaded patient category, which depends on several other factors in addition to the general body iron load including drug efficacy, organ targeting, toxicity aspects and compliance parameters [83,122,147]. There are also many controversies and biases in the selection and use of chelating drugs for optimal therapy, which go beyond iron overload [83,87,152]. Similarly, in most cases there is also no consensus in the evaluation criteria and risk/benefit assessment for the use of each of the chelating drugs in personalised medicine [123,128]. In particular, in the developing countries where most patients live, the main barrier to effective chelation therapy is the cost of the chelating drugs and their availability [83,87,142,143].

**Figure 2:**
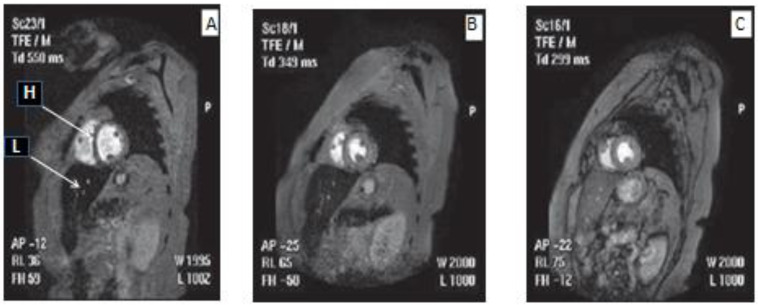
Clearing of excess iron from the heart and the liver of an iron-loaded thalassemia patient using the deferiprone and deferoxamine ICOC combination therapy protocol. The pictures show the magnetic resonance imaging (MRI) changes during the deferiprone/deferoxamine ICOC combination therapy. Short axis view of liver (L) and heart (H) of an iron-loaded male thalassemia patient (38 years, 69 Kg) at 5 months before the deferiprone/deferoxamine combination (Picture (**A**): Cardiac T2* was 14.5 ms and liver T2* 3.7 ms. Serum ferritin was 1626 μg/L, (estimated 1 month after the MRI scan); 9 months after the combination (Picture (**B**): Cardiac T2* was 17 ms and liver T2* 8.4 ms. Serum ferritin was 686 μg/L, (estimated 5 months after the MRI scan); and twenty and a half months after the combination, where normal iron levels have been achieved (Picture (**C**): Cardiac T2* was 20.7 ms and liver T2* 18 ms. Serum ferritin was 186 μg/L, (estimated 2.5 months after the MRI scan). ICOC: International Committee on Chelation T: signal intensity relaxation time. MRI: magnetic resonance imaging. Adapted with permission from reference [159].

The diagnosis and monitoring of EID are crucial parameters in the design of chelation therapy protocols for different categories of iron-loaded patients [81,131]. The impact of different chelation protocols on gross body iron load, as well as on individual organ iron load levels has been achieved mainly following the introduction of the MRI T2 and T2* diagnostic procedures. These methods have increased our understanding of iron metabolic and chelation pathways in iron removal and resulted in improved drug targeting therapies of gross body iron load, as well as focal iron deposits in organs, which are the cause of toxic side effects [81,131,144,153,154,155]. Most importantly, the use of MRI T2* monitoring has increased the prospects of the introduction of personalised medicine in TM and other iron overload metabolic disorders [156,157,158]. In particular, MRI T2* in combination with serum ferritin was used to monitor the efficacy of different chelating drugs and chelation protocols, and especially the International Committee on Chelation (ICOC) protocols of L1 or the L1/DF combination, which have been shown to achieve the complete elimination of excess iron from the heart and liver in TM patients of different levels of EDI (Figure 2) [144,159,160,161,162,163]. 

Negative iron balance and clearance of excess iron in both the heart and the liver has been shown in most TM cases using the ICOC protocol of oral L1 (75–100 mg/kg/day) and subcutaneous DF (40–60 mg/kg/day at least 3 days per week) (Figure 2) [144]. In such cases, the clearance of excess iron from these organs has been shown to be achieved within 0.5 to 1.5 years of commencing treatment, and to be faster when using higher overall doses of L1 and DF and in patients who are less heavily iron-loaded [159,160,161]. In contrast, excess iron removal from the heart by DFRA is ineffective and particularly slow, especially in cases of heavily iron-loaded TM patients [164,165]. In such cases, excess, toxic amounts of iron still remain in the heart of TM patients even after 5 years of treatment with DFRA at doses of 30–40 mg/kg/day [165,166]. Excess iron removal from the liver by DFRA appears to be effective usually at high doses and equivalent to that achieved by DF and L1 and less in comparison to their combination [159,160,161,166]. 

The most effective and rapid method of clearance of excess iron from the liver, which is also used for intensive chelation in heavily iron-loaded patients is intravenous DF in combination with oral L1 at the maximum tolerated doses. Much lower overall doses and in particular L1 monotherapy are used following the normalisation of the iron stores in the liver and heart, which is usually characterised by normal levels of MRI T2 and T2* and also serum ferritin (Figure 2) [161,162]. At this stage, the compliance of the patients is also much higher and the cost of chelation therapy much lower in comparison to other categories of more heavily iron-loaded patients. Most importantly, there is a substantial improvement in the quality of life of TM patients with normal iron stores [136,138].

## 4. Toxicity Concerns and Limitations in the Use of Iron-Chelating Drugs

Transfusional iron-loaded patients are regularly monitored for iron toxicity, chelating and other drug toxicity, as well for adverse effects caused by RBC transfusions and other pathological effects of their underlying condition. Similarly, regular clinical examinations and biochemical tests are carried out for organ function, including liver enzyme levels, echocardiography, serum iron and zinc levels, etc. [122,132,136].

The treatment of iron overload in various categories of patients with various iron load levels requires the selection of different chelation protocols for reducing the prospect of iron toxicity in heavily iron-loaded patients but also conversely of chelator toxicity in patients with low iron stores [136,146]. Regular monitoring and prophylactic measures for chelating drug toxicity are also generally recommended for the latter category of patients [132,136,167]. For example, the treatment of patients of iron-loading conditions with serum ferritin lower than 500 μg/L is restricted for DFRA due to toxicity implications, as described by the labelling drug information for its use by the manufacturing company [168,169]. This limitation is important for the safety of many regularly RBC-transfused patients, including young or other patients who have a small number of RBC transfusions [167,168,169,170].

Several toxic side effects such as renal, liver and bone marrow failure including agranulocytosis, as well as renal toxicity, skin rashes, gastric intolerance, etc., have been reported in iron-loaded patients treated with DFRA (Table 1) [167,170,171]. The toxicity of DFRA is even higher in patients with non-iron-loaded conditions, where increased morbidity and mortality have been reported at the initial period of its launching [170,171,172]. Furthermore, patients treated with DFRA are regularly monitored for kidney disfunction and withdrawal of the drug is recommended for patients with increases in serum creatinine levels [136,168,169].

Many of the limitations and restrictions which apply in the use of DFRA in non-iron-loaded patients also apply for the use of DF, despite the fact that the incidence of serious toxic side effects is much lower in the latter [173]. In this context, the use of DF in non-heavily iron-loaded categories of patients, as well as other categories of patients with normal iron stores is restricted due to DF toxicity implications [173]. For example, fatal mucormycosis has been reported in renal dialysis patients with normal iron stores treated with DF [173,174]. Furthermore, ocular and auditory toxicity has been reported in non-heavily iron-loaded TM and other categories of patients using DF [173,175,176]. 

In contrast to DF and DFRA, the relative safety of L1 in TM and other categories of iron-loaded patients with low or normal iron stores has been shown during the regular use of the drug in thousands of patients in the last 30 years and also in studies in TM patients with normal iron stores exceeding 100 patient years (Figure 2) [159,160,161,167]. The prospect of wider clinical use of L1 as a universal antioxidant in non-iron-overloading diseases such as neurodegenerative, cardiovascular, renal and infectious diseases and cancer has been investigated in clinical trials involving many of these categories of patients [112,138,167]. 

Clinical studies of L1 for the treatment of non-iron-loaded patients by targeting focal toxic EID, e.g., in Friedreich ataxia and also of toxic labile iron, e.g., in diabetic and non-diabetic glomerular disease are a reflection of the safety potential of this drug in non-iron-loaded conditions [112,138,167,177,178]. During these clinical studies, L1 was used at doses 50–75 mg/kg/day for 6–9 months with no serious toxic side effects [177,178]. Similarly, the safety of L1 in many other categories of non-iron-loaded diseases has also been confirmed in clinical trials involving patients with normal iron stores including the anemia of chronic disease, renal dialysis, infections, Parkinson’s and other neurodegenerative diseases [112,138]. 

The most serious toxic side effects reported for L1 include reversible agranulocytosis and neutropenia affecting less than 1% and 5% of patients, respectively (Table 1) [112,179]. Weekly or fortnightly mandatory blood count is recommended for patients using L1 for prophylaxis against this toxicity [136]. Several other less serious toxic side effects include gastric intolerance, joint and musculoskeletal pains and zinc deficiency [180,181]. In the latter case, zinc supplements are used for prophylaxis of TM patients on long-term treatment with L1 and also DF [84,136,181]. 

Similar regular monitoring of the progress of different therapies on the underlying conditions in all categories of iron-loaded patients in addition to chelation therapy is essential for their survival and quality of life [112,136]. In this context, toxicity vigilance and prophylactic measures are important parameters for ensuring safety, therapeutic outcome, long-term survival and reduction of morbidity and mortality in TM, TI, SCD and many other categories of iron-loaded patients treated with chelation therapy.

The efficacy of chelation therapy depends on many other factors and parameters associated with each patient, which appear to influence the rate of body iron intake, as well as the removal of excess iron from the body [122,136,182]. Such factors include clinical complications related to the age of patients, splenectomy, RBC antibodies and hemolysis, rate of dietary iron absorption, individual variations on chelating drug ADMET, chelating drug efficacy, compliance with treatment, infections, pregnancy, etc., (Table 1) [122,136,182]. For example, regular RBC transfusions can continue normally during pregnancy in TM patients but the use of iron chelation therapy by any of the three chelating drugs is not recommended because of the possibility of embryotoxicity. 

In the meantime, different chelation therapeutic approaches are emerging for various categories of transfusional iron-loaded patients [183,184,185]. For example, the prospect of early organ damage in young TM patients receiving RBC transfusions with an increase in liver, cardiac and pancreatic iron deposition was suggested following an MRI T2* study in a cohort of paediatric TM and other regularly transfused patients under 10 years of age [184]. Such findings suggest that chelation therapy should aim for the reduction and prevention of iron overload at young ages, or as soon as possible following about 20 units of RBC transfusions so that damage to organs from EID toxicity can be minimised, reversed and most importantly prevented (Figure 2) [81,183,184,185]. 

Despite the many circumstantial variations and factors contributing to iron overload toxicity in each category of transfused patients, every effort should be made to minimise associated organ damage and also to cause a decrease in the overall morbidity and mortality. In this context and similar to other diseases, personalised medicine based on individualised, tailor-made, effective and safe chelation therapy protocols needs to be designed for treating the various categories of transfusional iron-loaded patients with different rates of body iron intake [118,186,187]. This procedure requires continuous adjustment of the iron chelation protocols until safely reaching the stage of negative iron balance and thereafter the achievement and maintenance of normal iron store levels (Figure 2) [118,159,161].

It has generally been shown that iron-loaded TM patients who have achieved normal iron stores are devoid of iron overload toxicity damage such as cardiomyopathy, liver fibrosis and cirrhosis, endocrine complications, etc. [81,118]. These latter toxic side effects are widely observed in heavily iron-loaded patients. An additional advantage in most patients achieving and maintaining normal iron levels is also that the iron overload toxicity complications are reversible [135,137,188]. Furthermore, the overall chelating drug doses are usually reduced for the maintenance of normal iron stores and the quality of life in this category of patients is highly improved. A general increase in the survival rate of TM patients is currently observed in many developed countries, mainly as a result of improved and more effective iron chelation therapy protocols [134,135,136,188]. Similar improved therapeutic results are also expected using effective chelation therapies in all other iron-loaded categories of patients receiving chronic RBC transfusions.

## 5. Effective and Optimal Iron Chelation Therapies in Iron-Loaded and Non-Iron-Loaded Patients 

Maximal effort is required for the selection and application of personalised, effective and optimal chelation therapies in iron-loaded patients. In each case, the selection of any chelation protocol is based on a risk/benefit assessment and for most iron-loaded patients all three chelating drugs may be used as monotherapies or combination therapies, with the latter being the more efficacious method for the rapid elimination of excess iron [118,134,135,136,137,163,189,190]. In many cases, chelating drugs can be switched over for patients not responding sufficiently or experiencing toxicity with one or more of the chelating drugs [118,190]. 

In general, the rate of reduction of EID in chronically transfused patients depends mainly on the initial body iron load, the rate of RBC transfusions and the efficacy, as well as the tolerance of the chelation therapy protocols. Despite the many complications, variations and factors involved in the excess iron intake and load, the ICOC and similar protocols appear to be generally effective and safe in most TM patients for progressively reducing excess iron load and reaching the stage of normal body iron store levels (Figure 2) [97,190,191]. In such cases, the individualised tailor-made chelation protocols, the continuous and close monitoring of the body iron stores, and the regular biochemical and clinical monitoring of the underlying disease pathology could ensure the long-term safety and well-being of the patients [118,136,162]. 

The paradigm of TM patients who have achieved and maintained normal iron store levels can also be implemented for all other categories of chronically RBC-transfused patients. In this context, the ICOC chelation protocols of the L1/DF combination and L1 monotherapy, as well as other similar protocols which can achieve and maintain normal iron stores, should be used as first-line chelation protocols for the treatment of all iron-loaded, chronically transfused patients (Figure 2). Similarly, the systematic monitoring and controlling of the stage of normal body iron store levels in ex-iron-loaded TM patients can also be used for the prevention of iron load in other categories of chronically RBC-transfused patients [117,118,122,136].

However, there is a minority of cases where the treatment of EID using the ICOC chelation protocol involving DF or L1 or their combination may not be feasible due to low tolerability or toxic side effects such as allergic reactions during parenteral infusion of DF or toxicity such as L1 agranulocytosis (Table 1). In such cases, optimal chelation protocols have to be designed based on DFRA [192]. Clinical studies have suggested that the use of DFRA as monotherapy or in combination with L1 and/or DF seems to stabilise the iron load in some categories of TM patients [87,192,193]. However, there is still no evidence that such therapies can be effective for accomplishing and maintaining normal iron store levels in TM patients. Similarly, there are serious concerns regarding toxicity and also the ability of such DFRA-based protocols for the rapid elimination of excess cardiac iron and the long-term survival of these patients.

Despite the fact that the emphasis was on chelation therapy for TM patients, there are many other categories of chronically transfused patients with a diverse genotypic and clinical picture including variable severity of the underlying disease and different levels of excess iron load in the liver, heart and other organs [81,82,83,84,85,86,120,121,183,184,185,189]. Similar approaches and limitations in relation to chelation therapy in TM could be translated to each of the other categories of chronically transfused patients [81,82,83,84,85,86,120,121,183,184,185,189]. For each patient case, the design and application of optimal chelation therapy protocols could be based on the rate of body iron intake from transfusions and the overall risk/benefit assessment in the use of the chelating drug (s), as well as other factors, such as those described for TM patients. For example, lower doses of L1 (25–75 mg/kg/day), and for shorter time periods than those used in TM patients, can achieve the normalization of the iron store levels in TI, Hb E/β-thalassemia and Hb H disease, where the rate of body iron intake is slower than in TM [81,87,194,195,196,197]. Similar findings from clinical studies using lower doses of DFRA (5–40 mg/kg/day) in patients with TI in comparison to TM were also observed, but with lower overall efficacy and higher toxicity [87,198,199]. In particular, the report of renal toxicity was a major aspect of the discontinuation of the clinical use of DFRA in some TM patients [200,201,202]. A further limitation in the use of DFRA in TI and similar categories of iron-loaded patients is the unlikely prospect of normalisation of body iron store levels, since there is a restriction on its use for patients with serum ferritin levels lower than 500 μg/L [168,169].

In contrast to the major success in transforming TM from a fatal to a chronic disease and achieving the elimination of all excess iron in different organs using a step-by-step approach, some investigators have been using different methodologies in neurodegenerative diseases. In particular, in one case the disappointing results in Parkinson’s disease patients were published by one of the world’s top medical journals in 2022 involving more than 60 authors, without questioning several problematic features of the study including the very low L1 posology (15 mg/kg) and dose protocol (15 mg/kg twice daily) and also the monitoring methodology [203]. Some of the drawbacks of the methodology used in this and other neurodegenerative disease trials is the lack of L1 metabolic studies and of iron metabolic balance studies at these very low L1 doses [203,204,205]. Furthermore, since the target was iron and EID sites in the brain, the rationale of the selection of low L1 doses, which could not greatly impact the level of EID or its complete elimination, raises questions about the validity of the concept and the aim of the study. Similar methodologies, drug doses and problematic findings were also observed for two other studies in Parkinson’s disease [203,204,205].

The selection of drug posology is a critical component for the evaluation of the risk/benefit assessment for the use of any drug in neurodegenerative or any other diseases [206,207,208]. In this context, it should be noted that previous dose escalation and iron metabolic balance studies have shown that the use of such low doses of L1 in iron-loaded and non-iron-loaded conditions were mostly insignificant and ineffective at increasing iron excretion levels or the improvement of other iron-related hematological parameters [149,209,210,211,212]. Overall, the rationale for dose protocol selection for the use of L1 or any other drug in any disease should be accompanied by pharmacological, toxicological and therapeutic evidence such as estimation of the pharmacokinetic, metabolic and other parameters, the critical drug concentration for optimal therapeutic activity, changes in the target organ of toxicity, etc., as previously shown in the case of the use of L1 in TM patients (Figure 2) [149,209,210,211,212]. 

It is interesting that some of the co-authors in the recent Parkinson’s disease study were also co-authors in another study involving non-iron-loaded categories of HIV-infected asymptomatic patients, where up to ten times higher doses of L1 have been used in comparison to those used in the recent Parkinson’s disease patient trial [203,204,205]. In the HIV study, seven HIV patients received 33 mg/kg three times daily, and seven HIV patients and six normal volunteers received a dose of L1 of 50 mg/kg three times daily [213]. It was also reported that 86% of the subjects involved in the clinical study tolerated the 99 mg/kg daily dose and 61% the 150 mg/kg daily dose. The rationale for using such high L1 doses came from previous in vitro findings suggesting that the L1 threshold at about 150 μM did not allow a viral breakthrough for up to 35 days on-drug and also at least 87 days off-drug for an HIV viral rebound. Overall, these studies have suggested that L1 was the first low-molecular-weight drug that offered the prospect of reducing the pool of cells that harbour infection-relevant HIV-1 DNA [213]. Several hematological, gastrointestinal and hepatobiliary adverse effects with primary toxicity and increase in serum liver enzymes were reported in the cohort of volunteers involved in the HIV trial [213].

A rationale for drug posology, including the estimation of minimum and maximum effective doses, based on in vitro, in vivo and clinical studies is critical for the risk/benefit assessment and therapeutic outcome in iron overload and all other diseases [66,87,112,122,149,213,214]. In each case, the design of key studies related to the efficacy of a drug and also long-term studies confirming its safety are essential for achieving maximum therapeutic outcomes, such as those accomplished with TM patients.

## 6. Drug Interactions and Metabolic Changes Affecting the Safety and Efficacy of Iron Chelation Therapy

In addition to the various factors and parameters influencing iron chelation therapy discussed in the previous sections, there are also many other areas in need of further investigation, which can play an important role in therapeutic outcomes and can affect the overall safety and efficacy of the iron-chelating drugs. In particular, drug interactions and metabolic effects may play an important role in iron chelation therapy and affect patients receiving certain foods or undergoing different therapeutic or diagnostic procedures.

In particular, the interactions of chelating drugs with essential and xenobiotic metals, reducing and oxidizing agents, natural and synthetic drugs or other molecules with metal-binding capacity can all play a major role in the therapeutic potential of iron chelation protocols. There are many examples of such interactions including the daily use of vitamin C in TM patients, which in combination with DF enhances iron excretion [210,215,216]. Similar enhancers of iron excretion in combination with DF have been suggested using combinations with the natural plant products silymarrin and curcumin and many other iron phytochelators including mimosine [217,218,219]. 

Interactions with essential metals such as zinc are also important for iron chelation. In this context, the prophylactic use of zinc supplementation is carried out to avoid zinc deficiency in iron-loaded patients treated long-term with DF and L1 [136,181]. In contrast, the prophylactic use of zinc supplementation was not sufficient to replace the loss of zinc in TM patients treated with DTPA, which was the major cause of abandonment of this drug for the treatment of iron overload [102,103,220]. Interactions with zinc, copper, aluminium and iron present in the gastrointestinal tract by DFRA and L1 are also expected during their oral administration, which subsequently can affect iron removal, as well as the overall therapeutic implications of these two drugs [80,221]. Although no increases in iron absorption were observed with L1, both the absorption of iron and aluminium from the gastrointestinal tract are likely to increase in the presence of DFRA due to the high lipophilicity of its metal complexes (Table 1) [150,151,167,168,169,170,171,172]. In contrast, DF inhibits both iron and aluminium gastrointestinal absorption and is used as an antidote in cases of poisoning by these metals [222].

The iron-chelating drugs are also expected to influence the metabolism of drugs involving iron and other metal complexes, e.g., maltol-iron used in iron deficiency, diagnostic metal complexes of, e.g., indium and technetium, theranostic metal complexes of, e.g., gallium, and therapeutic effects of metal complexes of, e.g., platinum, which are widely used in cancer chemotherapy [80,112,223,224]. It is proposed that in such cases the timing of iron chelation therapy should not coincide with the pharmacological activity of such metal complexes, which is likely to influence the efficacy and toxicity of both drug components. Similar interactions influencing the efficacy and toxicity of both iron-chelating and other drugs are expected from drugs with iron-chelating properties such as tetracycline and doxorubicin and food components containing, for example, different phytochelators [80,112,219].

Another major area of interactions of iron-chelating drugs in medicine influencing general therapeutic and toxicological activity is in relation to free radical pathology, which appears to involve almost all diseases [219]. This interaction is considered despite the fact that free radical pathology has not yet been recognized as a general medical condition, nor are any antioxidant or other drugs prescribed for its treatment [225]. However, the discovery in the last ten years of ferroptosis [226,227,228,229], a new programmed cell death process involving iron and found in almost all diseases including cancer [230,231,232,233], infectious [234], kidney [235] and cardiovascular diseases [236], Parkinson’s disease [237] and COVID-19 [238], has attracted the research interest of many clinical scientists towards iron metabolism and free radical pathology. During ferroptosis, highly reactive free radicals are generated by iron, which cause lipid peroxidation and damage to the cell membrane [226,227,228,229,230,231,232,233,234,235,236,237,238]. In this context, chelating drugs [239,240,241], other chelators, iron [242,243,244,245,246] and related metabolic pathways [247,248,249] and associated biomolecules [250,251,252,253,254,255] can play a major role in the modulation of ferroptosis and influence the treatment of all associated diseases. Similar considerations and interests are also developed by clinical scientists working on microbial diseases, where iron appears to be a major component and regulator of microbial growth, and in this context chelating drugs can play an important role in antimicrobial treatments [256,257,258,259,260].

The metabolic transformation of chelating and other drugs and the implication of their different metabolites on their overall efficacy and toxicity, including the chelating therapeutic activity on iron or other metals, is also an important area that needs further investigation. In this context, there are several key findings on the metabolism of chelating drugs which could facilitate the design of improved protocols and therapeutic outcomes and also the prospect of designing a new generation of chelating and other drugs based on the effects of metabolic changes.

**Figure 3:**
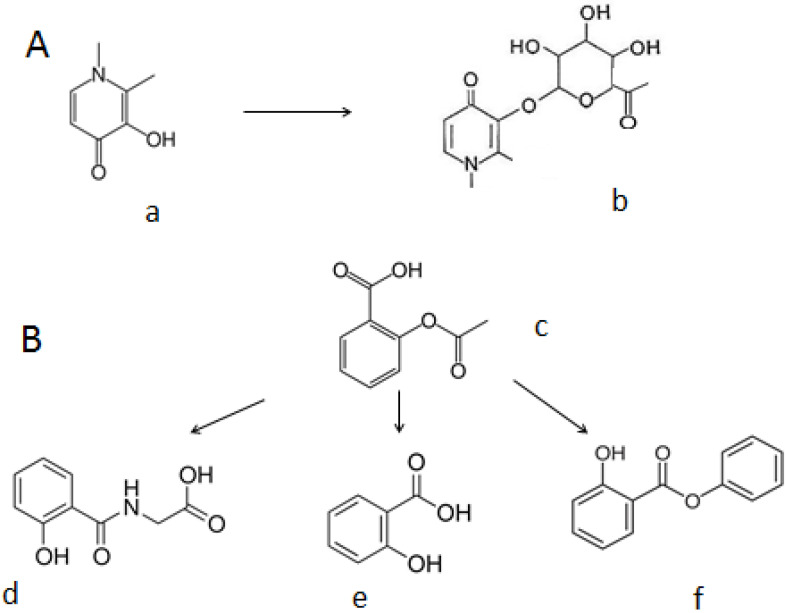
The metabolic transformation of deferiprone and aspirin. (**A**) Deferiprone (**a**) is metabolized to a glucuronide conjugate at the 3-OH group of the chelating site of deferiprone, forming deferiprone glucuronide (**b**), which has no iron-binding potential. (**B**) Aspirin (acetylsalicylic acid) (**c**) is metabolized to several metabolites including salicyluric acid (**d**), salicylic acid (**e**) and salicylic phenol (**f**), all of which have iron-binding affinities and form iron complexes (see related references [271,272,273]).

A characteristic example of metabolic effects is the chelator prodrug dexrazoxane, which is widely used in cancer patients for cardioprotection against doxorubicin and other anthracycline-induced cardiotoxicity, where iron is thought to be implicated in the toxicity [261,262,263,264,265,266,267]. Dexrazoxane is hydrolyzed in vivo to an EDTA-like active chelating compound ADR-925, which binds and removes the toxic form of iron involved in the cardiotoxicity [268,269,270]. In relation to iron-chelating drugs, DF has been reported to form several metabolites, some of which have chelating properties and may contribute to iron mobilisation and iron excretion [89,148]. There is no information as to which of the metabolite molecules of DF may cause any of the various adverse effects. In contrast, in the case of both L1 and DFRA their metabolic transformation to glucuronide conjugates abolishes the chelating potential of the drugs, and similarly their metabolites are not implicated in the toxic side effects of these chelating drugs (Table 1, Figure 3) [149,150,151]. Another variation in relation to chelation and metabolism is the case of aspirin (acetylsalicylic acid), where unlike the drug itself, its metabolites salicyluric acid, salicylic acid, salicylic phenol and 2,3-dihydroxybenzoic acid have all been found to have high affinity for iron binding and may affect iron metabolic processes (Figure 3) [271,272,273].

There are many other concepts in relation to the influence of drug interactions and metabolism on the overall efficacy and toxicity of iron chelation therapy. For example, the design of an orally active chelator entering the enterohepatic circulation and mobilising iron, which can then release the iron in the bile while the chelating drug is re-entering the circulation, may be ideal for long-lasting chelation therapy [274].

Overall, several other concepts for future investigations on chelating drugs are now more likely than in the past because of new developments and renewed interest in iron metabolism as a result of new discoveries such as ferroptosis, which appears to affect patients of all disease categories.

## 7. Future Prospects for the Design of Optimal Chelation Protocols in Patients with Excess Iron Toxicity

While the prospects of new developments in the area of iron chelation are expected to increase in the foreseeable future, the need for improved therapeutic approaches in existing patients with EID toxicity is imminent due to the overall high incidence of morbidity and mortality worldwide. In particular, new therapeutic strategies need to be introduced in each category of affected patients [81].

The variation in iron overload in different categories of chronically RBC-transfused patients and the restrictions imposed due to safety on the use of chelating drugs in such categories, as well as the need to target specific organs for the prevention of or recovery from iron load toxicity, require the design of effective or optimal chelation protocols in each case [81,118]. In this context, the selection of the most effective and safe chelation protocols, the close monitoring of iron load using serum ferritin and MRI T2*, and the use of prophylactic measures for preventing chelating drug toxicity could ensure the achievement of optimal chelation therapies including the effective reduction and maintenance thereafter of normal iron levels. This aim is, after all, one of the most important requirements for an overall decrease in the morbidity and mortality of all iron-loaded patient categories (Figure 2) [123,134,135,136,274,275,276].

Despite the major successes following the introduction of oral chelation and combination therapies that have transformed TM from a fatal to a chronic disease in many countries, there is a wide scope of improvements in other countries with lower survival rates in TM and also in many other categories of regularly RBC-transfused iron-loaded patients. One such important area which could facilitate iron chelation therapy in general is the design, development and availability of more effective chelating drugs and chelation protocols, which could increase the prospect of improved therapies for more categories of patients, especially those who have adverse reactions and cannot tolerate the monotherapies or combination therapies with L1, DF and DFRA. The introduction of new investigational chelating drugs, as well as their possible combination with clinically available chelating drugs, could benefit such categories of affected patients [66,277,278,279].

Another area of further improvement regarding iron chelation therapy is the possibility of development of naturally occurring chelators, especially ones from plants which can become readily available to patients (Table 1 and Table 2) [186,187,217,218]. One such natural chelator is mimosine, which could potentially be used as monotherapy or in combination therapies with other chelating drugs [219]. The availability of mimosine or other naturally occurring chelators could not only potentially benefit patients who cannot tolerate L1, DF and DFRA, but also the vast majority of TM patients living in developing countries who cannot afford the high prices of the synthetic chelating drugs [62,280,281,282]. Inexpensive synthetic drugs using new chemical synthesis of the already approved drugs may also benefit more patients [283].

There are several other categories of TM and other iron-loaded patients where different chelation strategies could be developed. Such strategies may include the design of specific chelators for oral administration for decreasing iron absorption, other routes of chelating drug administration such as DF suppositories, intravenous L1, chelating drug incorporation in the transfused blood, etc. [284,285,286]. Further strategies of improved chelation therapy could include the design of chelator pro-drugs such as dexrazoxane and aspirin and chelating drugs entering the entero-hepatic circulation as described in the previous section (Figure 3).

Different strategies in conjunction with chelation therapy could also be developed by reducing the rate of body iron intake from RBC transfusions, for example, through the reduction of RBC autoantibodies causing damage to transfused RBC [287,288]. Reduction of RBC transfusion requirements could also be achieved through improved transfusion methods involving the removal of old and damaged RBCs, increased induction of HbF production, introduction of antioxidants for the longer survival of RBCs before transfusion, improved spleen function, etc. [289,290,291,292,293].

New strategies for the reduction of body iron intake could also be developed based on dietary control of food iron absorption and also related pharmaceutical intervention with chelating and other drugs (Table 2) [284,292]. Similarly, the modulation of regulatory molecules and transcription factors involved in iron metabolism and erythropoiesis such as erythropoietin, hepcidin [294,295,296,297] and ferroportin [297,298], as well as other proteins and their combinations could also influence future therapeutic strategies involving iron chelation therapy (Table 2) [299,300,301,302,303,304].

The rate of iron overload could also be influenced by many and different clinical interventions. For example, the timing of initiation and rate of RBC transfusions as well as the timing of splenectomy could have a direct effect on the rate of iron loading and the adoption of new chelation strategies [293,305]. Furthermore, the intervention of different pharmaceutical preparations and dietary molecules [306,307,308,309,310,311,312], and different diseases and their treatment [313,314,315,316,317,318,319], could also influence iron absorption and excretion, as well as modulation of regulatory molecules involved in iron metabolism and erythropoiesis and the effect of chelation therapy [278,279,306,307,308,309,310,311,312,313,314,315,316,317,318,319,320,321].

Overall, several new and adjuvant treatments could be developed and become available for the future management of iron overload in chronically transfused patients. These could involve the use of new chelating drugs and optimal chelation therapy protocols for increasing iron excretion, selected diets and therapies for inhibiting iron absorption and the use of modulators for regulatory molecules involved in erythropoiesis and iron metabolism such as activators of HbF production, proteins affecting iron transport, utilisation and storage, as well as combinations of such treatments.

A global strategy is also required for the universal treatment of iron overload in all the different categories of chronically transfused patients including affected patients in developing countries, where chelation and other treatments are not currently available [62,81].

## 8. Conclusions

The risk/benefit assessment for the selection of appropriate drugs, drug protocols and effective, non-toxic drug doses is a dynamic process, which is built up from experiences gained from in vitro and in vivo studies as well as short- and long-term clinical studies. This drug evaluation process is critical for the therapeutic outcome of any disease, provided sufficient diagnostic methods are available for assessing the efficacy and toxicity of the drugs and drug protocols, as well as for the therapeutic progress or complete therapy of the patients treated. This step-by-step approach and incremental increase in posology was used in the case of chelation therapy in chronically RBC-transfused iron-loaded TM patients. In particular, the selection of specific chelating drugs including their combination at appropriate doses e.g., oral L1 (75–100 mg/kg/day) and subcutaneous DF (40–60 mg/kg/day at least 3 days per week) has been shown to cause the progressive removal of excess iron and the complete elimination of EID in different organs and especially the heart, which is the target organ of transfusional iron overload toxicity and the main affected organ contributing to the high mortality in TM. However, despite this major success and the progressive transition of TM from a fatal to a chronic disease in many developing countries, many problems still remain in TM and other transfusional iron-loaded conditions. These problems include drug toxicity; the unavailability of iron chelation therapy for TM and other categories of transfused patients in many developing countries due to scarce health resources; suboptimal posology information on optimal chelation therapies; the selection process of chelating drugs and protocols and risk/benefit assessment for some categories of patients with different underlying pathological complications; low response or toxicity of available chelation therapies in some patients; drug interactions; metabolic effects; etc.

In the meantime, following the discovery of ferroptosis and its association with most diseases, there is a renewed interest in the repurposing of chelating drugs as modulating drugs in such associated diseases. In this context, many efforts are also in progress for the improvement of iron chelation therapy including the design of new chelators, drug combinations, combination therapies with phytochelators, chelator pro-drugs and chelating drugs entering the entero-hepatic circulation. Overall, drug selection and the identification of effective or optimal dose protocols are essential parameters for positive therapeutic outcomes not only in the use of chelating drugs for TM, but also for all other diseases. Furthermore, a global strategy is required for the universal treatment of iron overload in all the different categories of chronically transfused patients including affected patients in developing countries with scarce health resources.

## Figures and Tables

**Table 1:** Summary of some properties of the iron-chelating drugs.

[A] CHEMICAL AND PHYSICOCHEMICAL PROPERTIES
Molecular weight of chelators: DF: 561. L1: 139. DFRA: 373.
Molecular weight of iron complexes: DF-Fe: 617. L1-(Fe)3: 470. DFRA-(Fe)2: 798.
Charge of chelators at pH 7.4: DF positive, L1 neutral, DFRA negative.
Charge of iron complexes at pH 7.4: DF positive, L1 neutral, DFRA negative.
Partition coefficient of chelators (n-octanol/water): DF: 0.02. L1: 0.19. DFRA: 6.3.
Stability constant (Log β) of chelator iron complexes at pH 7.4: DF-Fe: 31. L1-(Fe)3: 35. DFRA-(Fe)2: 27.
**[B] METABOLIC AND PHARMACOKINETIC PROPERTIES**
Metabolite(s): DF: A number of metabolites, cleared mainly through the urine, some with chelating properties.
L1: Gluguronide conjugate, cleared through the urine but has no iron chelation properties.
DFRA: Gluguronide conjugate, cleared through the feces and has no iron chelation properties.
T1/2 absorption following oral administration: L1: 0.7–32 min. DFRA: 1–2 h.
T max of the chelator: L1: Mostly within 1 h. DFRA: Mostly 4–6 h.
T1/2 elimination of chelator: Intravenous DF: 5–10 min. Oral L1: 47–134 min at 35–71 mg/kg. Oral DFRA: 19–6.5 h at 20 and 40 mg/kg.
T1/2 elimination of the iron complex: DF: 90 min. L1: Estimated within 47–134 min. DFRA: 17.2 ± 7.8 h at 20 mg/kg and 17.7 ± 5.1 h at 40 mg/kg.
T max of the iron complex: L1: Estimated within 1 h. DFRA: 1–6 h at 20 mg/kg and 4–8 h at 40 mg/kg.
T max of the metabolite L1-glucuronide: 1–3 h.
**[C] CLINICAL AND BIOLOGICAL EFFECTS**
Recommended doses of the chelating drugs in iron-loaded thalassemia patients: DF subcutaneously or intravenously 40–60 mg/kg/day. Oral L1 75–100 mg/kg/day. Oral DFRA 20–40 mg/kg/day.
Iron-loaded patient compliance with chelating drugs: Low compliance with parenteral DF in comparison to oral L1 and oral DFRA.
Iron removal from diferric transferrin in iron-loaded patients: About 40% at L1 concentrations >0.1 mM, but not by DF or DFRA.
Differential iron removal from various organs of iron-loaded patients: Efficacy is related to dose for all chelators. L1 preferential iron removal from the heart and DFRA from the liver. DF from the liver and less from the heart.
Increased excretion of metals other than iron: DF and L1 cause increased aluminium excretion in renal dialysis patients.
DFRA causes increases in aluminium and other toxic metal absorption.
Iron mobilisation and excretion of chelator metabolite iron complexes: Several DF metabolites have iron chelation potential and increase iron excretion but not the L1 glucuronide or the DFRA glucuronide metabolites.
Combination chelation therapy: L1, DF and DFRA combinations are more effective in iron excretion than monotherapy. The ICOC L1 and DF combination has been shown to cause normalisation of the iron stores in thalassemia patients.
Route of elimination of chelator and its iron complex: DF: Urine and feces. L1: urine. DFRA: Almost exclusively in feces and less than 0.1–8% in urine.
**[D] TOXICITY OF THE IRON-CHELATING DRUGS**
DF toxicity: Skin rashes and local reactions. Allergic and anaphylactic reactions. Ophthalmic toxicity. Auditory toxicity. Neurotoxic abnormalities. Pulmonary toxicity. Growth failure and bone abnormalities in children. Yersiniasis. Mucormycosis.
L1 toxicity: Agranulocytosis. Neutropenia. Musculoskeletal and joint pains. Gastric intolerance. Zinc deficiency.
DFRA toxicity: Renal, liver and bone marrow failure including agranulocytosis. Renal toxicity. Increases in serum creatinine levels. Skin rashes. Gastric intolerance.

Abbreviations: DF: deferoxamine, L1: deferiprone, DFRA: deferasirox, ICOC: International committee on chelation.

**Table 2:** Examples of factors and parameters affecting iron-chelating drug therapies.

Metal ion interactions with chelating drugs: Essential, xenobiotic, diagnostic and theranostic metals Chelating drug interactions: Natural dietary and biochemical molecules, drugs with chelating properties Oxidizing and reducing factors: Affect the redox properties of iron involved during chelation Metabolic factors: ADME parameters, chelating metabolites, enterohepatic circulation properties Different omic factors: Genomic, proteomic, metabolomic, pharmacogenomic, redoxomic, metallomic Efficacy factors: Selective targeting, monotherapy, combination therapies with drugs or natural chelators Risk/benefit assessment factors: Toxicity parameters from in vitro, in vivo and clinical studies Compliance factors: Route and duration of chelating drug administration, special requirement issues Therapeutic effect range: Fully effective, partly effective, non-effective therapies Drug posology range: Effective, optimal, sub-optimal, ineffective, placebo level Efficiency of diagnostic methods: Sufficient, partly sufficient, insufficient diagnostics Drug categories: Emergency, chronic, orphan drugs, nutraceuticals, folk medicines Drug role: Main drug, adjuvant therapy, antidote, synergistic effects, drug repurposing Drug administration: Oral, subcutaneous, intravenous, rectal, intramuscular Categories of patients affected: Iron-loaded, normal iron levels with EID, iron toxicity, iron metabolism Drug availability due to cost: Easy access in developed countries, limited access in developing countries Drug selection influences: Regulatory drug authorities, manufacturers, patient organizations, physicians Factors affecting iron absorption: Dietary molecules and medicinal preparations Therapeutic strategies in conjunction with chelation therapy: Induction of HbF production, antioxidants Therapeutic strategies involving chelating drug modulation of regulatory molecules of iron metabolism

ADME: absorption, distribution, metabolism, excretion. EID: Excess iron deposition.

## Abbreviations

ADME	absorption, distribution, metabolism and elimination
ADMET	absorption, distribution, metabolism, elimination and toxicity
DF	Deferoxamine
DFRA	Deferasirox
DTPA	diethylenetriaminepentaacetic acid
EDTA	ethylenediaminetetraacetic acid
EID	excess iron deposition
ICOC	International committee on chelation
L1	Deferiprone
MRI	magnetic resonance imaging
TM	Thalassemia major
TI	Thalassemia intermedia
SCD	Sickle cell disease
